# Comparative Efficacy of Three Minimally Invasive Procedures for Kümmell’s Disease: A Systematic Review and Network Meta-Analysis

**DOI:** 10.3389/fsurg.2022.893404

**Published:** 2022-06-01

**Authors:** Yajian Wang, Bo Liu, Zhenwei Sun, Yaning Zhang, Jiangping Su

**Affiliations:** Department of Orthopedics, Linfen People's Hospital, Linfen, China

**Keywords:** vertebroplasty, osteoporosis, vertebral compression fractures, Kümmell's disease, network meta-analysis

## Abstract

**Background:**

Percutaneous vertebroplasty (PVP), percutaneous kyphoplasty (PKP), and bone-filling mesh containers(BFC) are three viable minimally invasive techniques that have been used to treat Kümmell’s disease(KD). However, there is still debate as to which is safer and more effective. This study summarized the pros and cons of the three techniques in the treatment of KD through network meta-analysis(NMA).

**Methods:**

All eligible published clinical control studies comparing PVP, PKP, and BFC for KD up to December 2021 were collected by online search of Cochrane Library, PubMed, Embase, CNKI, Wanfang Database, and Chinese biomedical literature database. Data were extracted after screening, and Stata 16.0 software was used to perform the network meta-analysis.

**Results:**

Four randomized controlled trials (RCTs) and 16 retrospective case-control studies (CCTs) with a total of 1114 patients were included. The NMA results showed no statistical difference between the 3 procedures in terms of improving patients’ clinical symptoms. PKP was most likely to be the most effective in correcting kyphosis, while BFC was likely to be the most effective in managing the occurrence of cement leakage. No statistical differences were found in the incidence of new vertebral fractures in adjacent segments.

**Conclusions:**

Ranking analysis showed that BFC has the highest likelihood of being the optimal procedure for the treatment of KD, based on a combined assessment of effectiveness in improving patients’ symptoms and safety in the occurrence of adverse events.

## Introduction

Kümmell's disease (KD) is a form of delayed ischemic osteonecrosis following vertebral trauma, usually secondary to osteoporotic vertebral compression fractures (OVCFs) in the elderly (1). The radiological features are characterized by vertebral body collapse, intravertebral vacuum cleft (IVC), and pseudoarthrosis formation (2). Patients with KD tend to have intractable low back pain and severe kyphotic deformity. As the incidence of OVCF increases, the incidence of KD also tends to increase annually and can be as high as 12.1%–42.4% (3). Given that vertebrae with osteonecrosis fail to heal spontaneously, conservative treatment usually cannot yield satisfactory outcomes (4). In addition, there is a high risk of delayed neurological deficit following the vertebral collapse. Therefore, surgical treatments are highly recommended, especially for patients with intact neurological function, minimally invasive procedures are more popular (5, 6).

Percutaneous vertebroplasty (PVP), percutaneous kyphoplasty (PKP), and bone-filling mesh containers (BFC) are three minimally invasive procedures for the treatment of OVCFs in the elderly (7, 8). Likewise, their application in the treatment of KD has been gradually carried out in clinical practice with satisfactory outcomes (9). However, due to the specificity and complexity of KD, the application of minimally invasive techniques focuses not only on pain relief but also on preventing the deterioration of the deformity and the consequent nerve damage. In general, the concept of the minimally invasive procedure is to achieve treatment by delivering bone cement into the vertebral body (10). Specifically, PVP was performed by injecting bone cement into the compressed vertebral body under high pressure (11), whereas PKP was achieved by dilating the vertebral body with a balloon prior to cement injection (12). BFC was designed to control the dispersion of the bone cement within the vertebral body through a mesh container (13). Evidence from OVCFs showed that cement injection under high pressure can lead to associated complications, with bone cement leakage being the most common one (14). The presence of cleft within the vertebral body and even the formation of pseudo-articulations in KD implies a higher incidence of cement leakage. Therefore, how to reduce the incidence of cement leakage has become an unavoidable issue when choosing a treatment plan for KD.

To date, numerous studies have reported the clinical efficacy of the three procedures for the treatment of KD, but no consensus has been reached. To our knowledge, no study has systematically evaluated the advantages and disadvantages of these three minimally invasive procedures. Here, we collected the best available evidence to determine which approach has the highest effectiveness and fewest complications for KD by using a network meta-analysis (NMA), to provide useful evidence for clinical decision making.

## Materials and Methods

This study was conducted in accordance with the Preferred Reporting Items for systematic reviews and meta-analysis (PRISMA) guidelines (15) and the PRISMA NMA extension statement (16).

### Search Strategy

The literature retrieval was carried out by searching electronic databases including Pubmed, Embase, Cochrane Library, CNKI, and Wanfang Data. All relevant studies were retrieved from inception to December 2021. No limitations were applied to the language of publication. The keywords and mesh terms for the searching strategy were “Kümmell”, “vertebral osteonecrosis”, “vertebral pseudarthrosis”, “intravertebral vacuum cleft”, “vertebroplasty”, “PVP”, “percutaneous kyphoplasty”, “PKP”, “vesselplasty”, “bone-filling mesh container”, “compression fracture”, “OVCF”. A secondary search of the references of all eligible literature was also conducted to find additional papers omitted by the initial search strategy.

### Inclusion and Exclusion Criteria

Studies included in this review had to meet predefined criteria according to the PICOS approach (17). The inclusion criteria were as follows: (i). Patients: Adult patients with a clinical diagnosis of KD; (ii). Intervention: PVP, PKP, or BFC; (iii). Comparator: comparison of the effectiveness and safety of different treatment methods; (iv) Outcomes: visual analogue scale (VAS) score, Oswestry Disability Index (ODI), Cobb angle, cement leakage, and re-fracture of adjacent segments; (v). Study design: prospective randomized controlled trials (RCTs) or retrospective clinical control trials (CCTs). Studies meeting the following criteria were excluded: (i). Adult patients with a diagnosis of pathological fractures caused by primary or metastatic vertebral tumors; (ii). Single-armed studies without PVP/PKP/BFC as control, or cadaveric specimens/animals/biomechanical studies, or comparisons between high and low viscosity cements; (iii). Outcome evaluation did not include any of the above observations. (iv). Duplicate publications, reviews, conference abstracts, case series, letters, comments.

### Data Extraction and Literature Quality Assessment

Two reviewers (B.L. & Z.W.S.) independently screened all included studies. Data were extracted and put into a standardized form after reading the full text. Cross-checks were conducted to ensure consistency in the quality of literature extraction and analysis of results. Any apprehension encountered was resolved through discussions with a third person (Y.J.W.).

The methodological bias assessments of the included RCTs and CCTs were performed in compliance with the Cochrane risk of bias (RoB) tool (18) and risk of bias in non-randomized studies of interventions (ROBINS-I) tool (19), respectively. The average risk of bias contributions for each comparison within the network was shown with reference to the Confidence in Network Meta-Analysis(CINeMA) (20).

### Statistical Analysis

The analyses of NMA were completed and plotted using Stata 16.0 statistical software (StataCorp LLC, TX, USA). The results of the analysis of dichotomous variables were presented as relative risk (RR) or odds ratio (OR), and continuous variables were presented as weighted mean difference (WMD), both were expressed as 95% confidence interval (CI). For each observation, the inconsistency model was first applied, and the consistency model could be used for further analysis when the inconsistency test results were not significant (*p*-value >0.05). The node-splitting method was used to assess inconsistencies with both global and local to clarify the validity of direct and indirect comparisons. The surface under the cumulative ranking curve (SUCRA) was used to rank the superiority of different minimally invasive techniques. Forest plots were generated to show the relative risk and 95% CI of the outcomes of interest.

## Results

### Literature Search Results

**Figure 1:**
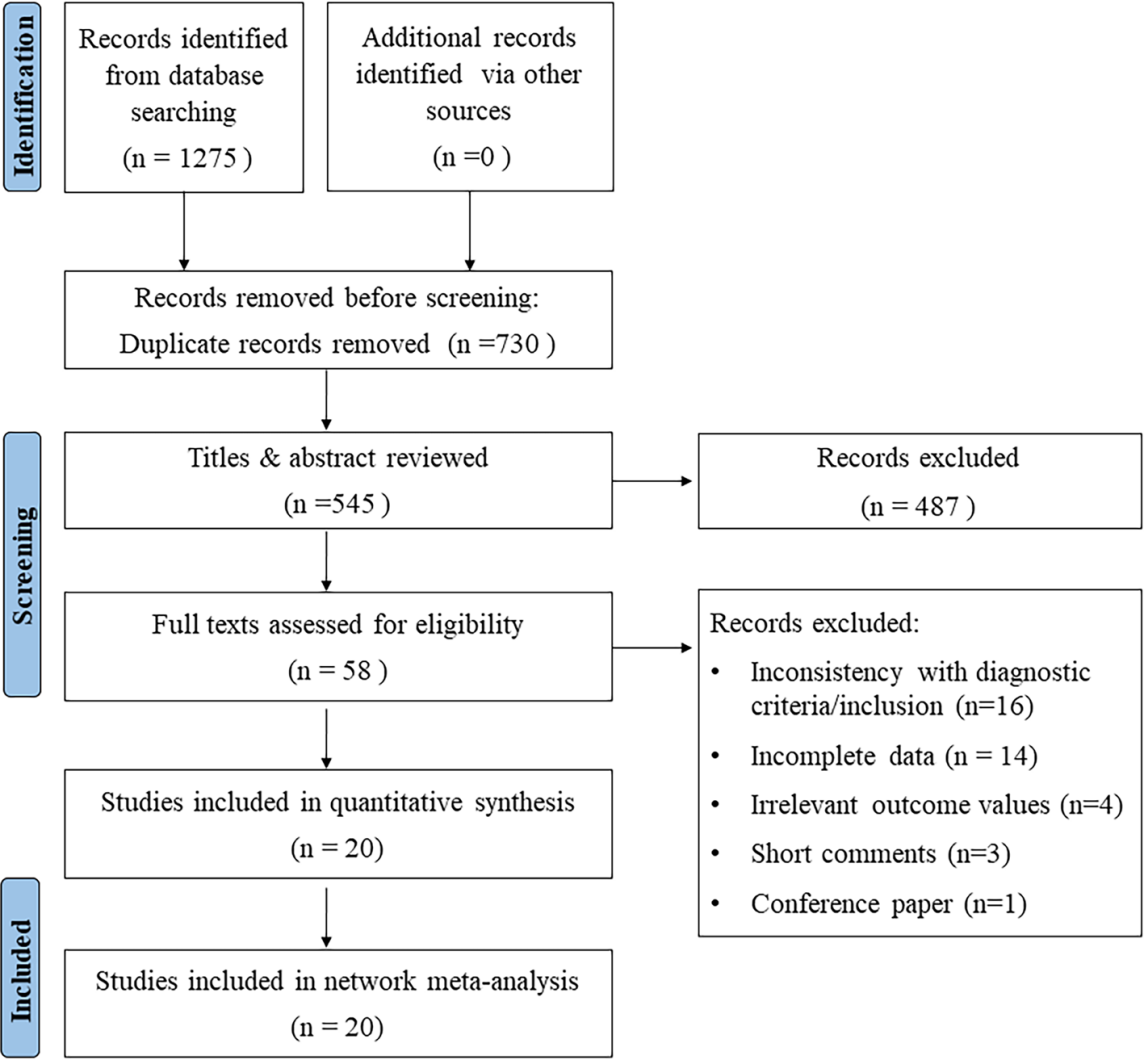
Flow diagram of the study identification and selection process.

A total of 1,275 studies were identified as potentially relevant by the search strategy. 730 duplicate or irrelevant studies were first removed. After screening the titles and abstracts, 487 ineligible studies were rejected. Based on the inclusion and exclusion criteria, 20 studies (21–40) were finally included for NMA after assessing the full text. The PRISMA flow chart for the literature selection was shown in Figure 1.

Four RCTs and 16 CCTs were identified, with a total of 1,114 patients. 438 (39.3%) received PVP, 496 (44.5%) had PKP, and 180 (16.2%) were treated with BFC. Study characteristics, patients’ demographics, and clinical data were shown in Table 1.

**Table 1:** The characteristics of included studies.

Studies	Design	Cases	Gender (M/F)		Age (years)				Procedures	Outcomes
Chen et al. (21)	CCT	33/30	4/29	3/27	69.2 ± 6.3		68.7 ± 6.5		PVP、PKP	a, d, e
Kong et al. (22)	CCT	24/29	8/16	7/22	70.5 ± 6.4		71.9 ± 7.0		PVP、PKP	a, b, c, d, e
Zhang et al. (23)	CCT	38/35	10/28	9/26	75.58 ± 4.97		73.74 ± 4.35		PVP、PKP	a, d, e
Gao et al. (24)	CCT	38/35	20/18	16/19	73 ± 6		75 ± 6		PVP、PKP	a, b, d
Yu et al. (25)	CCT	48/20	10/38	4/16	74.6		75.9		PVP、PKP	a, b, c, d, e
Yu et al. (26)	CCT	14/28	5/9	9/19	74.47 ± 5.79		71.56 ± 8.35		PVP、PKP	a, b, c, d
Zhang et al. (27)	CCT	22/13	7/15	5/8	72.82 ± 6.99		74.38 ± 5.66		PVP、PKP	d
Feng and Sun (28)	CCT	20/20	5/15	7/13	72.02 ± 4.96		72.43 ± 5.64		PVP、PKP	a, b, c, d
Yu et al. (29)	CCT	20/28	2/18	3/25	67.21 ± 4.57		67.05 ± 4.03		PVP、PKP	a, b, c, d, e
Wang et al. (30)	RCT	22/22	11/11	11/11	75.50 ± 5.00		74.90 ± 6.50		BFC、PKP	a, b, c, d
Xu et al. (31)	CCT	31/31	15/16	14/17	68.3 ± 1.9		67.9 ± 2.1		PVP、BFC	a, b, d
Duan et al. (32)	RCT	19/19	9/10	8/11	77 ± 5		76 ± 4		PVP、BFC	a, b, c, d, e
Han et al. (33)	CCT	31/31	13/18	11/20	71.3 ± 5.2		70.9 ± 6.4		BFC、PKP	a, b, c, d
Wan et al. (34)	CCT	23/18	17/24		74.5				BFC、PKP	d
Duan et al. (35)	RCT	20/20	9/11	8/12	>60		>60		BFC、PKP	b, c, d, e
Sun et al. (36)	CCT	28/35	8/20	13/22		70.3	71.5		BFC、PKP	a, b, c, d, e
Guo et al. (37)	CCT	38/6/30	17/21	3/3	14/16	65.3 ± 5.9	63.9 ± 4.8	66.5 ± 7.2	PVP、BFC、PKP	a, b, d
Chang et al. (38)	RCT	28/28	6/22	8/20	75.0 ± 5.8		75.1 ± 5.7		PVP、PKP	a, b, d, e
Yao et al. (39)	CCT	35/40	16/19	18/22	68.0 ± 3.6				PVP、PKP	a, b, c, d, e
Dai et al. (40)	CCT	30/34	9/21	12/22	75.81 ± 7.12		75.12 ± 6.92		PVP、PKP	d, e
										

***Note.*** *RCT, randomized controlled trials; CCT, retrieved clinical control trails; PVP, percutaneous vertebroplasty; PKP, Percutaneous kyphoplasty; BFC, bone-filling mesh containers; a, VAS scores; b, ODI scores; c, Cobb’s angle; d, cement leakage; e, adjacent segments re-fracture.*

### Risk of Bias Assessment

The assessments of the risk of methodological bias for RCTs and CCTs were shown in Supplementary Figures S1A, S1B, S2A,S2B, respectively. The average risk of bias contributions for each comparison within the network was summarized in Supplementary Figure S3.

### Effectiveness

#### VAS Score

A total of 16 studies reported postoperative VAS (21–26, 28–33, 36–39). The inconsistency model and the consistency model yielded consistent results(*p* = 0.43, Supplementary Figure S4). The node split analysis also showed the consistency of direct and indirect comparisons (Supplementary Table S1).

**Figure 2:**
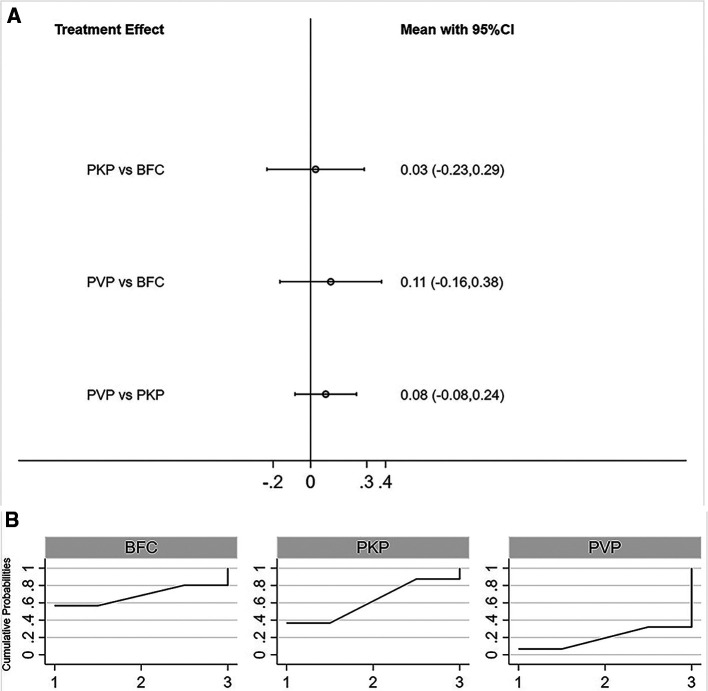
Forest plot of VAS score (**A**) and relevant SUCRA of each procedure (**B**). PVP, percutaneous vertebroplasty; PKP, Percutaneous kyphoplasty; BFC, bone-filling mesh containers.

All three procedures improved the patients’ postoperative VAS scores, but the differences were not statistically significant between any two procedures (Figure 2A). The SUCRA of each procedure were shown in Figure 2B. Based on this, the probability of obtaining the lowest VAS score was ranked, and the probability of BFC being the best option was 53.5% (Supplementary Table S2).

#### ODI Score

**Figure 3:**
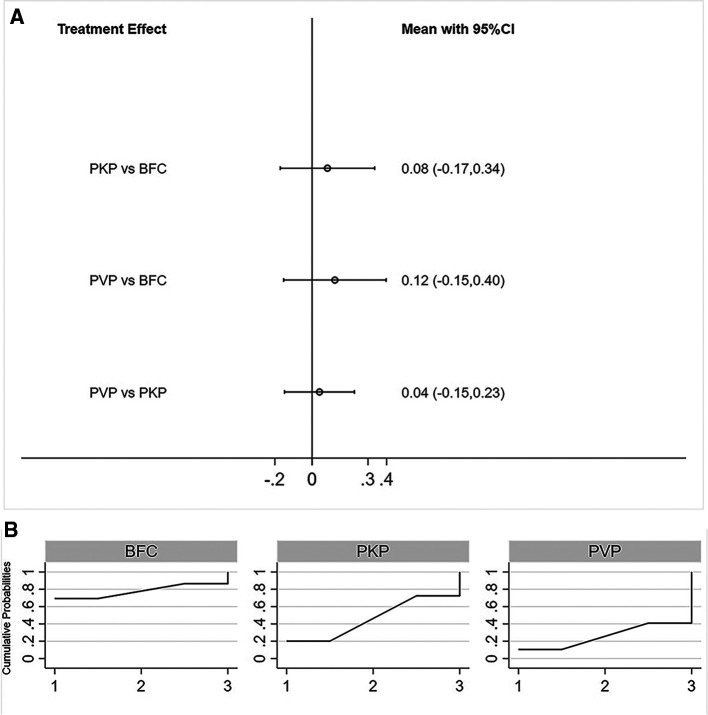
Forest plot of ODI score (**A**) and relevant SUCRA of each procedure (**B**). PVP, percutaneous vertebroplasty; PKP, Percutaneous kyphoplasty; BFC, bone-filling mesh containers.

Fifteen studies reported postoperative ODI scores for statistical analysis (22, 24–26, 28–33, 35–39). Consistency models and inconsistency models (*p* = 0.93, Supplementary Figure S5), direct and indirect comparisons (Supplementary Table S1), both yielded consistent results. All three procedures improved postoperative ODI scores, however, no statistically significant differences were found between any two procedures (Figure 3A). The SUCRA of each procedure were shown in Figure 3B. The probability ranking based on SUCRA showed that BFC had an 81.5% probability of being the best option for improving postoperative ODI (Supplementary Table S3).

#### Cobb Angle

**Figure 4:**
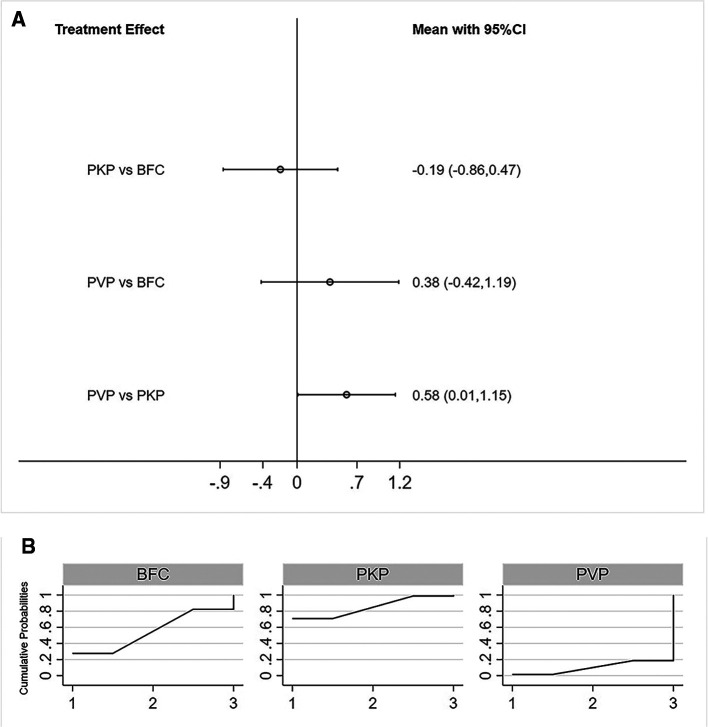
Forest plot of cobb angle (**A**) and relevant SUCRA of each procedure (**B**). PVP, percutaneous vertebroplasty; PKP, Percutaneous kyphoplasty; BFC, bone-filling mesh containers.

A total of 11 studies reported postoperative Cobb angle improvement (22, 25–26, 28–30, 32, 33, 35, 36, 39). Similarly, both consistency models vs inconsistency models (*p* = 0.41, Supplementary Figure S6), direct vs indirect comparisons (Supplementary Table S1), obtained consistent results. NMA results showed that PKP resulted in better postoperative Cobb angle improvement compared to PVP, neither PKP vs PVP, nor BFC vs PVP showed statistical difference(Figure 4A). The SUCRA of each procedure were shown in Figure 4B. The ranking results showed that the probability of PKP being the best procedure was 75.4% (Supplementary Table S4).

#### Cement Leakage

**Figure 5:**
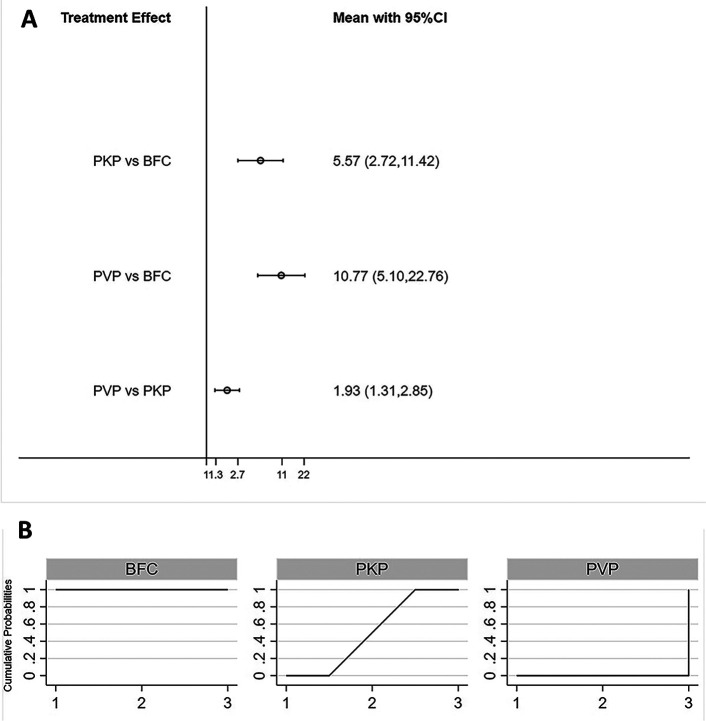
Forest plot of cement leakage (**A**) and relevant SUCRA of each procedure (**B**). PVP, percutaneous vertebroplasty; PKP, Percutaneous kyphoplasty; BFC, bone-filling mesh containers.

All 20 studies reported the incidence of bone cement leakage for statistical analysis (21–40). Consistency models and inconsistency models (*p* = 0.69, Supplementary Figure S7), direct and indirect comparisons (Supplementary Table S1), yielded consistent results. The incidence of bone cement leakage was ranked from low to high as BFC < PKP < PVP, with statistically significant differences between any two procedures(Figure 5A). The SUCRA of each procedure were shown in Figure 5B. The probability of BFC being the best procedure in terms of reducing the rate of cement leakage was 100% (Supplementary Table S5).

#### Adjacent Segments Re-Fracture

**Figure 6:**
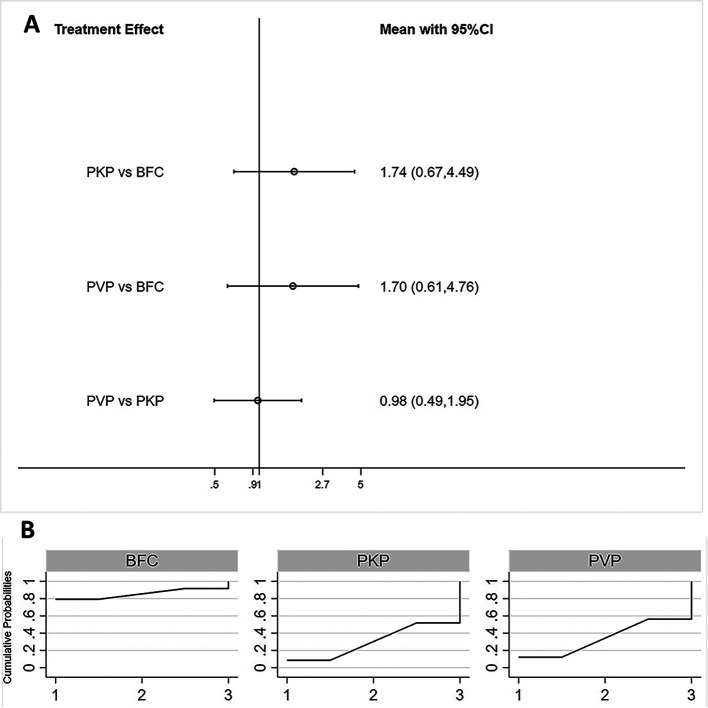
Forest plot of adjacent segments re-fracture (**A**) and relevant SUCRA of each procedure (**B**). PVP, percutaneous vertebroplasty; PKP, Percutaneous kyphoplasty; BFC, bone-filling mesh containers.

Eleven studies reported the incidence of re-fractures in adjacent segments for statistical analysis (21–23, 25, 29, 32, 36, 36, 38–40). Consistency models and inconsistency models(*p* = 0.86, Supplementary Figure S8), direct and indirect comparisons (Supplementary Table S1), yielded consistent results. There was no statistically significant difference in the incidence of postoperative re-fracture of adjacent segments among all three procedures (Figure 6A). The SUCRA of each procedure were shown in Figure 6B. The probability of BFC being the best procedure in terms of reducing the occurrence of adjacent segment re-fracture was 79.2% (Supplementary Table S6).

## Discussion

Compared to OVCFs, KD manifests as a rare and relatively complex spinal disorder, characterized mainly by greater vertebral instability, progressive deterioration, and more likely combined neurological deficit (41). Since the results of conservative treatment are usually limited, surgical treatment has become an option for more patients. For KD with neurological integrity, minimally invasive treatments are the preferred surgical approaches (42). With advances in disease understanding, the standardized diagnosis and therapeutic management of KD needs to be established with the support of high-level evidence. Numerous clinical studies have compared the clinical efficacy of PVP, PKP, and BFC for the treatment of KD (9, 35). Currently, the consensus is that all three minimally invasive procedures can improve patients’ symptoms, such as the relief of pain, improvement of functional status, and quality of life. However, there is still controversy regarding safety. It is inconclusive whether one procedure is better than the other in the treatment of KD. Therefore, we performed a network meta-analysis of the three minimally invasive procedures, ranking the likelihood of being the best procedure for each clinical outcome and presenting an objective and comprehensive view of their pros and cons.

With regard to the clinical outcomes, even after the addition of BFC, our findings were generally consistent with that of previous conventional meta-analysis which directly compared PVP vs PKP for the treatment of KD (9). That was, no statistical difference was found among the three procedures in terms of VAS and ODI scores, but PKP provided better kyphosis correction than PVP. This was attributed to the balloon expansion effect related to PKP (43). In the case of BFC, although homogeneous diffusion of the bone cement was achieved by the mesh container, it did not show the advantage of correcting the deformity over PVP. However, it demonstrated a definite advantage over the other two procedures in terms of preventing cement leakage. PVP performed the worst outcome in terms of bone cement leakage management. A meta-analysis showed that the incidence of cement leakage was as high as 54.7% and 18.4% for PVP and PKP, respectively (44). There is no doubt that the application of BFC offered a new option for the prevention of bone cement leakage.

In addition, other attempts have been carried out to optimize injection protocols to reduce bone cement leakage. Taking the most economical PVP as an example, surgeons have developed a sequential infusion of bone cement was prepared into a late-phase filiform or early-phase mass shape (similar to high-viscosity bone cement) and injected slowly, followed by the infusion of the bone cement as an early- or midphase filiform shape. By doing so the bone cement leakage rate can be reduced from 41.7% to 14.3% (45). This was similar to the outcome of treating KD with a high-viscosity bone cement product, which reduced the cement leakage rate from 45.2% to 13.6% (46). Moreover, studies have been conducted to modify the composition of bone cements to better match the biological and biomechanical characteristics of the human body. The addition of mineralized collagen to bone cement has been shown to have similar clinical efficacy as traditional bone cement and can reduce the incidence of bone cement leakage (47). The results of these new technologies are promising, and we look forward to more clinical studies based on these new technologies to comprehensively assess their clinical applicability. Meanwhile, regarding the issue of cement leakage, there is no doubt that it has the potential to lead to catastrophic consequences. However, we would like to have more discussion about this. We think more attention should be paid to how many cement leaks are true “adverse events,” i.e., what type of cement leak occurred, whether it led to new clinical symptoms, and what percentage of early or late revisions resulted from it. On this basis, we believe it is more meaningful to compare the pros and cons of the different procedures, but unfortunately, we were not able to obtain enough information from the included literature for a comprehensive analysis, and we expect new studies to cover the details of adverse events.

Undeniably, this study has some limitations. It is well known that the lack of large sample size, multicenter, prospective randomized controlled trials is a common gap in current clinical studies. When RCTs are not sufficient to answer the question of interest, nonrandomized studies can be included for meta-analysis (48). Sixteen of the enrolled studies we included were CCTs, which may have a low-quality grade due to lack of randomization and blinding, as well as the possibility of greater potential bias. When we evaluated the methodological quality of these studies using the ROBINS-I tool, their measurement of outcomes and selection of the reported results were both at high risk, implying a possible overestimation of treatment effects. Therefore, this needs to be taken into account when referring to the results of this study. There is no doubt that more prospective multicenter RCTs with long-term follow-up are needed to evaluate the clinical efficacy of the three procedures for KD. In addition, the study locations included were all in China, or rather, this study tended to reveal the effectiveness of these three minimally invasive procedures for the treatment of KD in the Chinese population. The good point is that we performed NMA, an advanced form of meta-analysis, to integrate and compare both direct and indirect evidence from clinical studies to inform clinical decisions by means of a ranked manner (49). To the best of our knowledge, this is the first NMA evaluating minimally invasive approaches for the treatment of KD.

In conclusion, this NMA performed a hierarchical ranking of the effectiveness and safety of three minimally invasive procedures for patients with KD. The ranking analysis showed that BFC had the highest likelihood of being the best procedure for the treatment of KD based on a combined assessment of effectiveness in improving patient symptoms and safety in the occurrence of adverse events. Our findings present new evidence for the selection of minimally invasive treatments for KD, providing surgeons with informative support in clinical practice, decision making, and guideline designation.

## Data Availability

The original contributions presented in the study are included in the article/Supplementary Material, further inquiries can be directed to the corresponding author/s.
