# Supplementary material for: Comparative Efficacy of Three Minimally Invasive Procedures for Kümmell’s Disease: A Systematic Review and Network Meta-Analysis
Source: Front Surg. 2022 Jun 1;9:893404. doi: 10.3389/fsurg.2022.893404 (PMC9198435; doi:10.3389/fsurg.2022.893404)
Supplement: Supplementary file 1 [file Data_Sheet_1_v1.docx]

**Table S1. Node-splitting analysis results of direct and indirect comparisons**

**Note.** PVP: percutaneous vertebroplasty; PKP: Percutaneous kyphoplasty; BFC: bone-filling mesh containers.

| **Comparisons** | **p-value** | | | | |
| --- | --- | --- | --- | --- | --- |
|  | **VAS** | **ODI** | **Cobb angle** | **Cement leakage** | **Adjacent segments re-fracture** |
| **BFC, PKP** | 0.09 | 0.69 | 0.41 | 0.98 | 0.86 |
| **BFC, PVP** | 0.10 | 0.62 | 0.41 | 0.75 | 0.86 |
| **PKP, PVP** | 0.08 | 0.53 | 0.41 | 0.57 | 0.86 |

**Table S2. Rank possibility of VAS**

**Note.** PVP: percutaneous vertebroplasty; PKP: Percutaneous kyphoplasty; BFC: bone-filling mesh containers. SUCRA: surface under the cumulative ranking curve.

| **Procedures** | **SUCRA** | **PrBest** | **Rank** |
| --- | --- | --- | --- |
| **BFC** | 68.6 | 56.7 | 1 |
| **PKP** | 62.1 | 36.6 | 2 |
| **PVP** | 19.4 | 6.7 | 3 |

**Table S3. Rank possibility of ODI**

**Note.** PVP: percutaneous vertebroplasty; PKP: Percutaneous kyphoplasty; BFC: bone-filling mesh containers. SUCRA: surface under the cumulative ranking curve.

| **Procedures** | **SUCRA** | **PrBest** | **Rank** |
| --- | --- | --- | --- |
| **BFC** | 78.0 | 69.4 | 1 |
| **PKP** | 46.3 | 20.1 | 2 |
| **PVP** | 25.7 | 10.5 | 3 |

**Table S4. Rank possibility of Cobb angle**

**Note.** PVP: percutaneous vertebroplasty; PKP: Percutaneous kyphoplasty; BFC: bone-filling mesh containers. SUCRA: surface under the cumulative ranking curve.

| **Procedures** | **SUCRA** | **PrBest** | **Rank** |
| --- | --- | --- | --- |
| **BFC** | 55.0 | 27.6 | 2 |
| **PKP** | 84.9 | 70.9 | 1 |
| **PVP** | 10.1 | 1.5 | 3 |

**Table S5**. **Rank possibility of cement leakage**

**Note.** PVP: percutaneous vertebroplasty; PKP: Percutaneous kyphoplasty; BFC: bone-filling mesh containers. SUCRA: surface under the cumulative ranking curve.

| **Procedures** | **SUCRA** | **PrBest** | **Rank** |
| --- | --- | --- | --- |
| **BFC** | 100.0 | 100.0 | 1 |
| **PKP** | 50.0 | 0.0 | 2 |
| **PVP** | 0.0 | 0.0 | 3 |

**Table S6. Rank possibility of adjacent segments re-fracture**

**Note.** PVP: percutaneous vertebroplasty; PKP: Percutaneous kyphoplasty; BFC: bone-filling mesh containers. SUCRA: surface under the cumulative ranking curve.

| **Procedures** | **SUCRA** | **PrBest** | **Rank** |
| --- | --- | --- | --- |
| **BFC** | 85.5 | 79.3 | 1 |
| **PKP** | 30.3 | 8.6 | 3 |
| **PVP** | 34.2 | 12.1 | 2 |
